# Overview of female healthcare providers’ outlooks on cervical cancer screening: a narrative review

**DOI:** 10.3389/fonc.2025.1496513

**Published:** 2025-02-03

**Authors:** Wubishet Gezimu, Ababo Demeke, Firomsa Bekele, Solomon Seyife Alemu, Lema Fikadu, Addisalem Workie Demsash, Geleta Nenko Dube, Gemeda Wakgari Kitil, Elias Ezo

**Affiliations:** ^1^ Department of Nursing, College of Health Sciences, Mattu University, Mattu, Ethiopia; ^2^ Department of Nursing, College of Health Sciences, Dilla University, Dilla, Ethiopia; ^3^ Department of Pharmacy, Institute of Health Science, Wallaga University, Nekemte, Ethiopia; ^4^ Department of Midwifery, Maddawalabu University, Shashemene, Ethiopia; ^5^ Department of Midwifery, Institute of Health Science, Wallaga University, Nekemte, Ethiopia; ^6^ Department of Health Informatics, Debre Berhan University, Debre Birhan, Ethiopia; ^7^ Department of Health Informatics, College of Health Sciences, Mattu University, Mattu, Ethiopia; ^8^ Department of Midwifery, College of Health Sciences, Mattu University, Mattu, Ethiopia; ^9^ Department of Nursing, College of Health Sciences, Wachemo University, Hossana, Ethiopia

**Keywords:** cervical cancer, screening, knowledge, barriers, female healthcare providers, narrative review

## Abstract

**Background:**

Cervical cancer is the second most frequent gynecologic cancer. Uniquely, it is easily preventable and treatable cancer if identified early. The insights of healthcare providers about cervical cancer screening have a crucial role in prevention and treatment. However, there has been limited literature on the providers’ perspectives on cervical cancer screening.

**Objective:**

This review narrated the female healthcare providers’ (FHCPs’) outlooks on cervical cancer screening in terms of risk perceptions, awareness, knowledge, attitude, practice, and possible barriers.

**Methods:**

A thorough literature search was conducted to identify studies conducted on female healthcare providers’ overview of the perceived risk of cervical cancer, cervical cancer screening awareness, knowledge, attitude, and practice, as well as barriers to cervical cancer screening. Databases such as PubMed, Medline, Embase, Virtual Health Library, and Google Scholar were used to search for articles.

**Results:**

Accordingly, this review identified that female healthcare providers have a low perceived risk of the disease, poor awareness and knowledge, unfavorable attitudes, and low uptake of screening practices. Furthermore, this review highlights the obstacles to cervical cancer screening acceptance, such as service inaccessibility, a lack of training and education, and fear of the procedure and results.

**Conclusion:**

This narrative review described the variable distribution of the FHCPs’ perceived risk of acquiring cervical cancer (CC). Poor knowledge and screening practices were observed. Moreover, the barriers to cervical cancer screening uptake were described. Given that healthcare providers are on the frontlines (act as role models) in increasing the community’s cervical cancer screening uptake, we suggest concerned bodies increase screening access and implement staff training programs. In addition, further mixed studies should be considered to deeply understand the possible attributes ingrained in individual and social belief systems.

## Introduction

Globally, approximately 9.2 and 4.4 million new cancer cases and deaths were recorded in the female population in 2020, respectively. Cervical cancer (CC) was found to be the most commonly diagnosed gynaecologic cancer and the second leading cause of death in the same year, following only breast cancer (1). Additionally, CC gravely disturbs the survivor’s quality of life (2).

In comparison to that in technologically developed countries, the burden of CC is higher in developing countries (1, 3). Poor regions of the world, including Sub-Saharan Africa (SSA), were particularly hard hit by CC, with 90% of cases reported (4). According to GLOBOCAN 2020, CC accounted for approximately 5,338 deaths in Ethiopia (5). In the same country, it was also the most common cause of cancer death in most reproductive-age women (15–44 years) (6).

CC is mostly caused by the human papillomavirus (HPV) (7). Globally, approximately 70% of all CC cases are caused by the high-risk (oncogenic) strains, HPV-16 and HPV-18, and the rest of the cases are caused by strains such as 31, 33, 45, 52, and 58 (4, 8, 9). According to the American Cancer Society’s guidelines, this cancer-causing virus can be prevented by strategies such as risk reduction, being vaccinated, and undergoing screening timely (10).

CC is a tumour that can be easily prevented and treated if detected early (8). In resource-poor settings, HPV vaccination and screening are effective and profitable options for eliminating CC (11). Cervical cancer screening (CCS) can easily detect precancerous cells. The HPV test, the Pap test, and visual inspection with acetic acid are all methods for detecting CC (12). Screening is recommended for all women aged 21 to 65 years. According to the American Academy of Family Physicians and the U.S. Preventive Services Task Force, the interval for CCS is every 3 years for women aged 21 to 29 and every 5 years for women aged 30 to 65 years (13).

Although it is a preventable cancer type, approximately one-half of women with CC had not undergone screening before diagnosis (13). Remarkably, screening and case treatment are underutilized in resource-limited settings where CC accounts for 90% of case fatalities (4).

The World Health Organization (WHO) set a 90-70-90 target for resourced-limited countries by 2030, with the goal of reaching 90% HPV vaccination of girls by the age of 15, 70% HPV screening by the age of 35, and 90% treatment of women diagnosed with the disease by the age of 45 (14).

Healthcare providers are the major sources of health information for clients and the general public (15, 16). According to a study by F. Kimondo, H. Kajoka, M. Mwantake, et al., approximately 80% of CCS was conducted by healthcare providers (17). Healthcare providers are pioneers whose beliefs, attitudes, and approaches linearly affect their clients’ intention and utilization of the service they provide. They have a professional obligation to educate, motivate, and promote screening and other preventative measures to their clients. They are the clients’ main sources of information about risk factors, prevention, and treatment of CC (18–21). Thus, recognizing their awareness, knowledge, attitude, and barriers to screening has a fundamental role in the prevention and management of CC. This review narrates the female healthcare providers’ (FHCPs’) outlooks on cervical cancer screening.

## Methods

We conducted an overview of the literature, which is one type of the three narrative literature reviews identified by B. Green, C. Johnson, and A. Adams (22). Three authors (WG, AD, and FB) executed a thorough literature search for 1 week (from February 2 to 18, 2022) using advanced search strategies for all important studies published up to the last date of the search. The search included different databases such as PubMed, Medline, Embase, and Virtual Health Library. Additionally, we rigorously searched Google Scholar and government databases to access reports and unpublished studies. We connected the search terms such as “female”, “woman”, “Health extension worker”, “Healthcare provider”, “healthcare Professional”, “Healthcare worker”, “physician”, “doctor”, “nurse”, “midwife”, “radiologist”, “pharmacist”, “dieticians”, “medical” “laboratory technician”, “dentists”, “physiotherapists”, “optometrist”, “occupational therapist”, and “physician assistant” using Boolean operators including OR and AND. After completion of searches, we retrieved and saved all the search results in Mendeley Library.

### Article selection criteria

In this review, we included all the global literature (regardless of geography and publication period) of studies that were conducted on female healthcare providers’ overview of the perceived risk of CC, CCS awareness, knowledge, attitude, and practice, as well as barriers to cervical cancer screening. We excluded from this review the articles not accessed in full length and those published in languages other than English.

### Quality assessment

Four authors (WG, AWD, GND, and EE) conducted a quality check for the retrieved articles based on their relevance to our predetermined topics of interest, whether the outcome was appropriately identified, and methodological thoroughness.

## Results and discussion

### Perceived risk of the disease

All the articles that assessed FHCPs’ perceived risk of disease were on studies conducted in the South and Southeast Asian countries. For instance, in Singapore, 98% of female nurses had a perceived risk of CC, which is tied to adequate knowledge of the cancer (23). According to a study from Chennai, India, approximately 42% of FHCPs did not perceive that they were at risk for CC. Likewise, approximately 20% of providers had no intention to be screened (24). In a study conducted in Malaysia, a low perceived risk of CC is a barrier to screening (25). The perceived risk variations between populations from different countries could be due to personal beliefs, religion, perceptions, attitudes, and levels of knowledge about the disease.

### Awareness and knowledge of cervical cancer screening

The providers’ adequate CCS awareness could enhance the clients’ screening practice. Incompatible with this fact, the majority of the literature shows poor awareness among FHCPs. For instance, a study conducted by A. Med., D. Hastanesi, A. Hekimli, et al. revealed low awareness of CCS among FHCPs (20). A study conducted in Malaysia also showed FHCPs’ low awareness of CCS (25). Moreover, FHCPs’ low CCS awareness was observed in Ethiopia, one of the SSA countries (26, 27). Contrary to this, a study conducted by S. Sudharshini, V. Anantharaman, and A. Chitra found a higher level (95%) of CCS awareness among FHCPs (24). The variability in the providers’ awareness may be linked to curricular variations and training availability. Hence, a mixed-approach study is suggested to explore such attributes of awareness.

Unlike that of the clients, healthcare practitioners’ inadequate knowledge has a far-reaching influence on the entire community. To improve screening behavior, women’s understanding of the risk factors, causes, early indications, and treatment choices for CC is critical (28). Undoubtedly, in the case of healthcare providers’ inadequate knowledge, it is difficult to empower the community’s screening behavior and awareness. In this review, healthcare providers had limited knowledge of CCS. A study conducted by B. Obeidat, Z. Amarin, and L. Alzaghal identified FHCPs’ poor awareness of screening in Jordan (29). A study conducted in Saudi Arabia revealed that only 4% of FHCPs had a good level of knowledge (30). In a study from Nigeria, approximately 71% of FHCPs had poor knowledge of CCS (31). A study conducted in Ethiopia found more than one-half of FHCPs had poor knowledge (27). Similarly, in a study conducted in Turkey and Jordan, more than one-half of the participants had poor knowledge (18, 20). A study conducted on nursing staff in India revealed that 77% of participants knew about CCS (32). In contrast, in a study conducted in Albania, more than three-fourths of FHCPs had sufficient knowledge of CCS (33). These variable distributions of providers’ screening knowledge could be associated with exposure to capacity-building training and academic curricular variations.

### Attitudes toward cervical cancer screening

Providers’ attitude toward screening is another important factor in increasing the awareness and practice of individual clients. In a research conducted in Saudi Arabia, approximately three-quarters of FHCPs believed that screening is useful in preventing CC (30). A study conducted in Ethiopia found that only one-quarter of FHCPs supported CCS (27). Generally, we found too little literature on this specific topic of interest. Hence, further quantitative and qualitative research on this population is necessary to construct strong evidence.

### Cervical cancer screening practices

Evidence about CCS practice FHCPs was reviewed from 10 articles. A study from Jordan found that 80% of FHCPs had never been screened (29). In Singapore, less than one-half of nurses had never undergone CCS (23). A study from Chennai, India, revealed that 82% of FHCPs have never undergone screening for CC (24). A. Med., D. Hastanesi, A. Hekimli, et al. also identified healthcare providers’ poor screening practices (20). In Saudi Arabia, only one-fourth of FHCPs have been screened (30). A study conducted by M. Urasa and E. Darj showed that 85% of participants had not undergone screening at all, and the majority did not even know the intervals of CCS (34). Similarly, a survey from India found that 85% of nurses had never been screened (32). A survey conducted in South-South Nigeria revealed that 89% of healthcare workers had never been screened (31). K. Fatjona, G. Theodhosi, Y. Bilushi, et al. revealed that more than three-fourths of FHCPs had not ever practiced CCS (33). Moreover, approximately 91% of FHCPs had not undergone screening in Ethiopia (27). In general, most articles reviewed in this study showed poor CCS practice among FHCPs, where more than three-fourths had not undergone screening. This screening practice gap may be explained by different underlying factors, including privacy issues (being screened by a staff member), fear of procedures and positive results, poor risk perception, and attitudes toward the disease.

### Barriers to cervical cancer screening

In this review, evidence is gathered on the inaccessibility of services, fear of the procedures and results of screening, and lack of health education and training as factors that hinder FHCPs from screening.

### Inaccessibility of services

CC commonly affects women who live in low- and middle-income countries (LMICs) that are deprived of resources for prevention and treatment (35). The current review supports this fact. All articles that explored inaccessibility as a constraint for screening were identified to be from LMICs. Accordingly, a qualitative study from Malaysia explored the lack of resources as a main barrier to screening uptake (25). A study conducted in Jordan showed that more than one-half of FHCPs had not been screened due to a lack of screening services (29). N. Haweissa, J. Lim, and T. Su identified that limited accessibility was due to the expensive cost of screening Libya (19). This problem is exceptionally high in Sub-Saharan Africa (35). The absence of screening kits and inadequate rooms in facilities were stated as barriers to CCS as indicated by evidence from Ethiopia (21, 27, 36). Another study conducted in Ethiopia revealed that a lack of screening materials and infrastructures hinders users from screening utilization (26).

### Lack of health education and training

Poor health information affects the disease prevention and treatment behavior of an individual (37). In Tanzania, M. Urasa and E. Darj found that approximately 85% of participants reported the need for health education in their workplace (34). Lack of in-service training has been identified as a factor affecting screening knowledge. In Albania, insufficient staff training was reported as a hindering factor for screening service uptake by healthcare providers (33). In a study conducted in Ethiopia, only 16% of participants have undergone in-service training (36). This fact is also supported by our previous study (27). The studies from Jordan and Ethiopia showed that the likelihood of screening uptake of healthcare providers was higher among those who have undergone training (27, 29).

### Fear of the procedure and screening result

The client’s perception of pain during the screening procedure, according to evidence, hinders them from screening (38). According to the findings of S. Sudharshini, V. Anantharaman, and A. Chitra, FHCPs had not undergone screening due to embarrassment and diffidence (24). C. Yong, L. Hong, K. Lee, and colleagues hypothesized that participants found screening painful and distressing (25). Moreover, a study conducted in Singapore showed that nurses’ false perception of pain was a reason for non-utilization of screening (23). According to a study conducted in Tanzania, 9.5% and 7.3% of nurses denied being screened due to fear of the procedure and the results, respectively (34). Moreover, G. Eze, I. Obiebi, and I. Umuago identified fear of screening procedures as a reason for not undergoing screening (31).

### Limitations

This review sheds light on the scientific understanding of CCS from the providers’ perspective, particularly from female healthcare providers, which has been poorly researched in the field. However, since we conducted a narrative review that did not strictly follow a systematic process, it may lack methodological rigor and reproducibility. Hence, we suggest that researchers in the field consider systematic reviews, meta-analyses, and qualitative approaches to exploring deep personal and societal beliefs.

## Conclusion

Factually, the majority of scientific communities and clinicians have been working on boosting the CCS insights of the users. We thought that the providers’ own insight and practice are fundamental to boosting the user’s knowledge, attitude, and screening practice. This narrative review described the variable distribution of the FHCPs’ perceived risk of acquiring CC. Unexpectedly, poor knowledge and screening practices were observed among the FHCPs. In addition, the review also presented barriers to CC screening uptake among FHCPs, including service inaccessibility, a lack of training and education, and fear of screening methods and screening results. Given that healthcare providers are on the frontlines (act as role models) in increasing the community’s CCS uptake, we suggest concerned institutions increase screening access and implement staff training programs. In addition, further mixed studies should be considered to deeply understand the possible attributes ingrained in individual and social belief systems.

## References

[1] Sung H Ferlay J Siegel RL Laversanne M Soerjomataram I Jemal A. Global cancer statistics 2020: GLOBOCAN estimates of incidence and mortality worldwide for 36 cancers in 185 countries. CA Cancer J Clin 2. (2021) 71:209–49. doi: 10.3322/caac.21660 33538338

[2] Rutherford C Tait M Mileshkin L King MT. Quality of life in women with cervical cancer. Uterine Cervical Cancer: Clin Ther Perspect. (2019), 267–89.

[3] Ahmed S. Ureterocalycostomy in the management of renal and ureteric trauma: report of a case. Aust N Z J Surg. (1976) 46:381–2. doi: 10.1111/j.1445-2197.1976.tb03253.x 1071564

[4] Martin JY Erickson BK Huh WK. World health organization regional office for Africa: cervical cancer. Evidence-Based Obstet Gynecol. (2018), 165–72.

[5] World Health Organization. Ethiopia, globocan 2020. Int Agency Reseach Cancer. (2021) 133:2020–1.

[6] Bruni L Albero G Serrano B Mena M Collado JJ Gómez D. Human papillomavirus and related diseases in Ethiopia. Summary report . Available online at: www.hpvcentre.com. (Accessed February 2, 2022).

[7] World Health Organization. United Nations Population Fund. Preparing for the introduction of HPV vaccines: policy and programme guidance for countries(2006). Available online at: http://whqlibdoc.who.int/hq/2006/WHO_RHR_06.11_eng.pdf. (Accessed February 2, 2022).

[8] Centers for Disease Control. Inside Knowledge about Gynecologic Cancer: Cervical cancer(2019). Available online at: cdc.gov/cancer/knowledge800-CDC-INFO. (Accessed February 2, 2022).

[9] Centers for Disease Control and Prevention. Cancers associated with human papillomavirus, United States—2014–2018 USCS data brief, no. 26 Vol. 26). . Atlanta, GA: Centers for Disease Control and Prevention, US Department of Health and Human Services (2021) p. 25–6.

[10] American Cancer Society medical and editorial content team. Cervical cancer causes, risk factors, and prevention(2020). Available online at: www.cancer.org/cancer/acs-medical-content-and-news-staff.html. (Accessed February 2, 2022).

[11] Sankaranarayanan R. Screening for cancer in low- and middle-income countries. Ann Glob Heal. (2014) 80:412–7. doi: 10.1016/j.aogh.2014.09.014 25512156

[12] World Health Organization. Comprehensive Cervical Cancer Control, A guide to essential practice. 2nd ed. Geneva: World Health Organization (2014) p. 366–78. 25642554

[13] Rerucha CM Darnall CR Medical A Hood F. Cervical cancer screening. Am Fam Physician. (2018) 97:441–8. www.aafp.org/afp. 29671553

[14] World Health Organization. Global strategy to accelerate the elimination of cervical cancer as a public health problem and its associated goals and targets for the period 2020 – 2030 Vol. 2. Geneva: United Nations General Assembly (2020) p. 1–3 p.

[15] Ruddies F Gizaw M Teka B Thies S Wienke A Kaufmann AM. Cervical cancer screening in rural Ethiopia: A cross- sectional knowledge, attitude and practice study. BMC Cancer. (2020) 20:1–10. doi: 10.1186/s12885-020-07060-4 PMC7298871 32552740

[16] Al-Amro SQ Gharaibeh MK Oweis AI. Factors associated with cervical cancer screening uptake: Implications for the health of women in Jordan. Infect Dis Obstet Gynecol. (2020). doi: 10.1155/2020/9690473 PMC7114762 32256030

[17] Kimondo FC Kajoka HD Mwantake MR Amour C Mboya IB. Knowledge, attitude, and practice of cervical cancer screening among women living with HIV in the Kilimanjaro region, northern Tanzania. Cancer Rep. (2021) 4:1–8. doi: 10.1002/cnr2.1374 PMC8551985 33739611

[18] Ifemelumma CC Anikwe CC Okorochukwu BC Onu FA Obuna JA Ejikeme BN. Cervical cancer screening: assessment of perception and utilization of services among health workers in low resource setting. Int J Reprod Med. (2019) 2019. doi: 10.1155/2019/6505482 PMC6377970 30854395

[19] Hweissa NA Lim JNW Su TT. Health-care providers’ perceptions, attitudes towards and recommendation practice of cervical cancer screening. Eur J Cancer Care (Engl). (2016) 25:864–70. doi: 10.1111/ecc.2016.25.issue-5 27350095

[20] Med A Hastanesi D Hekimli A Dal A Kad H Sa H. Knowledge, attitudes and behaviors of health workers about cancer screenings. Ankara Med J. (2017) 17:73–83. doi: 10.17098/amj.304666

[21] Vu M Yu J Awolude OA Chuang L. Cervical cancer worldwide. Curr Probl Cancer. (2018) 42:457–65. doi: 10.1016/j.currproblcancer.2018.06.003 30064936

[22] Green BN Johnson CD Adams A. Writing narrative literature reviews for peer-reviewed journals: secrets of the trade. Clin Updat. (2001) 5:101–17. doi: 10.1016/S0899-3467(07)60142 PMC2647067 19674681

[23] Tay K Tay SK Tesalona KC Rashid NMR Tai EYS Najib SJM. Factors affecting the uptake of cervical cancer screening among nurses in Singapore. Int J Gynecol Obstet. (2015) 130:230–4. doi: 10.1016/j.ijgo.2015.03.037 26032624

[24] Sudharshini S Anantharaman V Chitra A. A cross-sectional study on knowledge, attitude, and practice on cervical cancer and screening among female health care providers of Chennai corporation, 2013. J Acad Med Sci. (2012) 2:124. doi: 10.4103/2249-4855.141132

[25] Yong CJ Hong LL Lee KY Krishnasamy I Nasir NHB Gravitt P. Healthcare providers´ Views on cervical screening: A qualitative study of barriers to cervical screening in Malaysia. J Glob Oncol. (2018) 4:214s–s. doi: 10.1200/jgo.18.86300

[26] Lott BE Halkiyo A Kassa DW Kebede T Dedefo A Ehiri J. Health workers’ perspectives on barriers and facilitators to implementing a new national cervical cancer screening program in Ethiopia. BMC Womens Health. (2021) 21:1–14. doi: 10.1186/s12905-021-01331-3 33941159 PMC8090515

[27] Abebaw E Tesfa M Gezimu W Bekele F Duguma A. Female healthcare providers ‘ knowledge, attitude, and practice towards cervical cancer screening and associated factors in public hospitals of Northwest Ethiopia. SAGE Open Med. (2022) 10. doi: 10.1177/20503121221095931 PMC9118899 35600715

[28] Cervical cancer control in developing countries: Memorandum from WHO meeting. Bull World Health Organ. (1996) 74:345–51. PMC2486879 8823955

[29] Obeidat BR Amarin ZO Alzaghal L. Awareness, practice and attitude to cervical Papanicolaou smear among female health care workers in Jordan. Eur J Cancer Care (Engl). (2012) 21:372–6. doi: 10.1111/j.1365-2354.2011.01297.x 22050559

[30] Heena H Durrani S Alfayyad I Riaz M Tabasim R Parvez G. Knowledge, Attitudes, and Practices towards Cervical Cancer and Screening amongst Female Healthcare Professionals: A Cross-Sectional Study. J Oncol. (2019) 2019. doi: 10.1155/2019/5423130 PMC6854973 31772579

[31] Eze GU Obiebi IP Umuago IJ. Perspectives of cervical cancer and screening practices among staff of a teaching hospital in South-South Nigeria. J Cancer Res Pract. (2018) 5:67–73. doi: 10.1016/j.jcrpr.2018.01.001

[32] Shekhar S Sharma C Thakur S Raina N. Cervical cancer screening: Knowledge, attitude and practices among nursing staff in a tertiary level teaching institution of rural India. Asian Pacific J Cancer Prev. (2013) 14:3641–5. doi: 10.7314/APJCP.2013.14.6.3641 23886159

[33] Fatjona K Theodhosi G Bilushi Y Cuberi D Sinaj E. An overview of cervical cancer knowledge and screening among female healthcare practitioners. Eur Sci J. (2020) 10(30).

[34] Urasa M Darj E. Knowledge of cervical cancer and screening practices of nurses at a regional hospital in Tanzania. Afr Health Sci. (2011) 11. PMC3092321 21572857

[35] Sankaranarayanan R Budukh AM Rajkumar R. Effective screening programmes for cervical cancer in low- and middle-income developing countries. Bull World Health Organ. (2001) 79:954–62. PMC2566667 11693978

[36] Burrowes S Holcombe SJ Leshargie CT Hernandez A Ho A Galivan M. Perceptions of cervical cancer care among Ethiopian women and their providers: a qualitative study. Reprod Health. (2022) 19(1):1–19. doi: 10.1186/s12978-021-01316-3 34983586 PMC8725313

[37] Zimmerman E Woolf SH. Understanding the relationship between education and health(2014). Available online at: http://www.iom.edu/understandingtherelationship. (Accessed February 5, 2022).

[38] Binka C Nyarko SH Awusabo-Asare K Doku DT. Knowledge towards cervical cancer prevention and screening practices among women who attended reproductive and child health clinic at Magu district hospital, Lake Zone Tanzania: A cross-sectional study. BMC Can BioMed Res Int. (2019) 2019:1–8. doi: 10.1186/s12885-018-4490-7 PMC5956852 29769124

